# Multi omics analysis of fibrotic kidneys in two mouse models

**DOI:** 10.1038/s41597-019-0095-5

**Published:** 2019-06-14

**Authors:** Mira Pavkovic, Lorena Pantano, Cory V. Gerlach, Sergine Brutus, Sarah A. Boswell, Robert A. Everley, Jagesh V. Shah, Shannan H. Sui, Vishal S. Vaidya

**Affiliations:** 1 Laboratory of Systems Pharmacology, Harvard Medical School, Boston, MA USA; 2 Department of Medicine – Renal Division, Brigham and Women’s Hospital, Boston, MA USA; 3 Bioinformatics Core, Harvard T. H. Chan School of Public Health, Boston, MA USA; 4 Department of Environmental Health, Harvard T. H. Chan School of Public Health, Boston, MA USA; 5 Department of Systems Biology, Harvard Medical School, Boston, MA USA

**Keywords:** miRNAs, Biomarkers, Target identification, Proteomics, Transcriptomics

## Abstract

Kidney fibrosis represents an urgent unmet clinical need due to the lack of effective therapies and an inadequate understanding of the molecular pathogenesis. We have generated a comprehensive and combined multi-omics dataset (proteomics, mRNA and small RNA transcriptomics) of fibrotic kidneys that is searchable through a user-friendly web application: http://hbcreports.med.harvard.edu/fmm/. Two commonly used mouse models were utilized: a reversible chemical-induced injury model (folic acid (FA) induced nephropathy) and an irreversible surgically-induced fibrosis model (unilateral ureteral obstruction (UUO)). mRNA and small RNA sequencing, as well as 10-plex tandem mass tag (TMT) proteomics were performed with kidney samples from different time points over the course of fibrosis development. The bioinformatics workflow used to process, technically validate, and combine the single omics data will be described. In summary, we present temporal multi-omics data from fibrotic mouse kidneys that are accessible through an interrogation tool (Mouse Kidney Fibromics browser) to provide a searchable transcriptome and proteome for kidney fibrosis researchers.

## Abstract (summary)

**Table:** 

Design Type(s)	transcription profiling design • proteomic profiling design • stimulus or stress design
Measurement Type(s)	transcription profiling assay • protein expression profiling assay
Technology Type(s)	RNA sequencing • mass spectrometry
Factor Type(s)	experimental condition • temporal_instant • biological replicate
Sample Characteristic(s)	Mus musculus • kidney

Machine-accessible metadata file describing the reported data (ISA-Tab format)

## Background & Summary

More than 10 percent of adults in developed countries present with some degree of chronic kidney disease (CKD). One of the hallmarks of CKD is the development of fibrosis and subsequent renal failure. Mechanisms and pathways underlying the development of kidney fibrosis are still not widely understood and therefore treatment strategies are limited. Mouse models are frequently used to gain more insights into the fibrosis development and to evaluate potential drug candidates.

Two well-established mouse models for kidney fibrosis are folic acid (FA) induced nephropathy and unilateral ureteral obstruction (UUO)^1^. Both models display human relevant pathological tubulointerstitial fibrosis shortly after induction of injury, are easy to perform and have good reproducibility. Transcriptomics and proteomics studies for both models have been published before, mostly as a basis for follow-up experiments focusing on one selected gene/protein^2–7^. However, data for mRNA, miRNA and protein expression, including a combination of all three omics datasets, have not been generated in parallel for these models; therefore, we aimed to generate this comprehensive data to (1) characterize the UUO and FA model in depth, (2) allow integration of the three layers of omics data, and (3) provide a tool for hypothesis generation as well as testing.

**Fig. 1:**
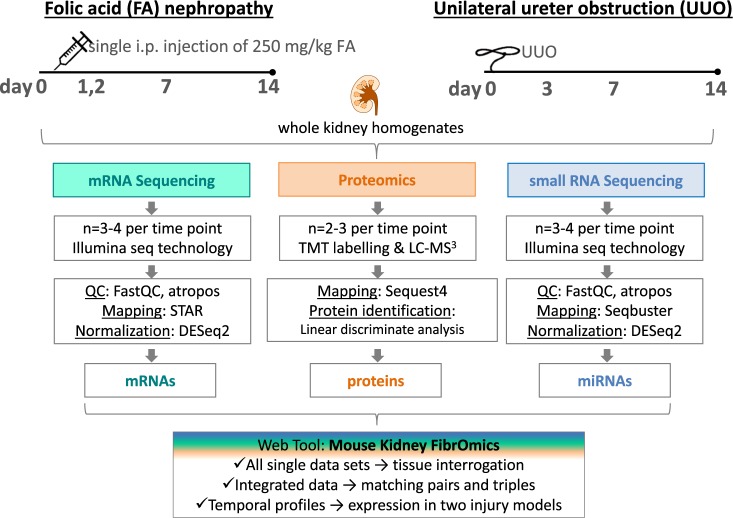
Schematic of study design, data generation and processing. Overview of how the kidney fibrosis models were set up including flow charts for mRNA-seq, proteomics and small RNA-seq profiling in the kidneys.

In the FA model, mice were sacrificed before the treatment (day 0) and 1, 2, 7, and 14 days after a single injection (250 mg/kg i.p.) of folic acid (Fig. 1). For the UUO model, mice were sacrificed before obstruction (day 0) and 3, 7, and 14 days after the ureter of the left kidney was obstructed via ligation (Fig. 1). For both studies, kidneys were removed at each time point for total RNA isolation and protein sample preparation. Total RNA was used for mRNA and small RNA sequencing on the Illumina platform. The transcriptomics data (mRNAs and miRNAs) from the FA model were previously published by our group^2,3^, but here we re-analysed the existing FA sequencing data in parallel with the newly generated UUO sequencing data, applying the same algorithms for consistency and inclusion. Proteomics in matching kidney samples from both models were measured by liquid chromatography/mass spectrometry (LC/MS) using 10-plex TMT. Temporal profiles were generated for each mRNA, miRNA and protein dataset in both models using day 0 as reference point. To combine all three sets, mRNAs and corresponding proteins were matched according to their annotations, whereas mRNAs and miRNAs were paired based on targets predicted using TargetScan.

For the UUO mRNA-seq data, an average of 30 million reads were sequenced, with 97% mapping to the transcriptome, less than 1% mapping to rRNA genes, and with 88% of the aligned reads mapping to exonic regions. For the UUO small RNA-seq data, an average of 20 million reads were sequenced, with 50% mapping to approximately 700 annotated miRNA genes.

In both proteomics datasets over 8,000 proteins were quantified, thus yielding overall a unique and comprehensive dataset of gene, protein and miRNA expression in the fibrotic mouse kidney.

Finally, all datasets can be viewed and interrogated by an online tool, the Mouse Kidney Fibromics browser: http://hbcreports.med.harvard.edu/fmm/.

## Methods

### Animal studies

Male BALC/c mice were obtained from Charles River Laboratories (USA). All experimental protocols concerning the use of laboratory animals were performed according to the NIH guidelines for the care and use of laboratory animals, and approved by the Institutional Animal Care and Use Committees (IACUC) of Harvard Medical School. Mice were housed in groups of three on a 12h light/dark cycle with access to food and water *ad libitum*. At the age of 8–10 weeks they entered the experiment.

#### Folic acid (FA) model

Folic acid was prepared at 25 mg/ml in 0.3 M sodium bicarbonate. Mice received a single dose of 250 mg/kg via intraperitoneal injection. Before the injection and 2, 7, and 14 days later, mice were sacrificed; kidneys were removed and immediately snap frozen in liquid nitrogen.

#### Unilateral ureter obstruction (UUO) model

Before surgery, mice were anaesthetized with an intraperitoneal injection of sodium pentobarbital (50 mg/kg of body weight), the left kidney was exposed via a flank incision and 3.0 silk suture thread was used to tie off the ureter at the lower pole. Before the obstruction and 3, 7, and 14 days later, mice were sacrificed; kidneys were removed and immediately snap frozen in liquid nitrogen.

More details about the *in vivo* experiments can be found elsewhere^8^.

### mRNA-seq: RNA extraction, library preparation and sequencing

#### Folic acid (FA) model

mRNA sequencing in kidneys from the FA model was published before^4^ (GSE65267)^9,10^. In brief, quantity and quality of isolated RNA were assayed on an Agilent 2200 TapeStation instrument and by SYBR qRT-PCR assay. 10 ng total RNA was used to prepare libraries with the IntegenX Apollo 324 system and NuGEN SPIA reagents. Libraries were multiplexed in groups of three per lane of a flow cell, and 50 cycles, paired-end sequencing was performed on an Illumina HiSeq2000 instrument.

#### Unilateral ureter obstruction (UUO) model

Total RNA was isolated using Qiagen’s RNeasy Mini Kit. Quality and quantity of the RNA was assessed photometrically and with the Bioanalyzer (Agilent). 330 ng total RNA were transcribed utilizing Illumina’s TruSeq Stranded mRNA Library Prep Kit. Libraries were pooled and sequenced on Illumina’s NextSeq500 as single end, 75 bp reads. Sequencing service was performed by the Molecular Biology Core Facilities at Dana-Farber Cancer Institute. Data were deposited to the Gene Expression Ombibus (GEO) database: GSE118339^9,11^.

### Small RNA-seq: RNA extraction, library preparation and sequencing

#### Folic acid (FA) model

small RNA sequencing in kidneys from the FA model was published before^3^ (GSE61328)^9,12^. In brief, total RNA was isolated using the miRNeasy Mini Kit (Qiagen). 1 µg total RNA was used to prepare small libraries utilizing the TruSeq Small RNA Sample Preparation Kit (Illumina) according to manufacturer instructions. All samples were multiplexed into a single lane of a flow cell on the HiSeq2000 platform to produce 50 cycles, single-end reads.

#### Unilateral ureter obstruction (UUO) model

Total RNA was isolated using Qiagen’s miRNeasy Mini Kit. Quality and quantity of the RNA was assessed photometrically and with the Bioanalyzer (Agilent). 1 µg total RNA was transcribed utilizing Illumina’s TruSeq Small RNA Library Prep Kit. Libraries were pooled and sequenced on Illumina’s NextSeq500 as single end, 75 bp reads. Sequencing service was performed by the Molecular Biology Core Facilities at Dana-Farber Cancer Institute. Data were deposited on GEO database: GSE118340^9,13^.

### Proteomics: protein sample preparation, TMT labelling and LC-MS3 measurement

Kidney samples (from both FA and UUO models) were mechanically homogenized in lysis buffer (8 M urea, 1% SDS, Roche complete protease inhibitors and phosphatase inhibitors, 50 mM Tris pH 8.5). Approximately one third of a kidney was used for sample preparation. Protein concentration was determined using the BCA assay (Pierce, Rockford, IL).

The homogenate was reduced with 5 mM DTT and alkylated with 15 mM iodoacetamide (Sigma, St. Louis, MO). 0.15 mg of protein was precipitated using chloroform:methanol. Pellets were washed twice with cold methanol and re-solubilized in 8 M urea with 20 mM EPPS, pH 8.5. After diluting the samples to 4 M urea using 20 mM EPPS, they were digested with Lys-C (Wako Chemicals, Richmond, VA) overnight at room temperature. On the next day, samples were further diluted to 1.5 M urea using 20 mM EPPS and digested for 6h at 37 °C using Trypsin (Promega, Madison, WI). 60 µg of each sample were then brought to 10% (v/v) acetonitrile and labeled with 2:1 (TMT:Peptide) by mass of TMT-10 reagent (Pierce). The reaction was quenched with hydroxylamine (0.5% final volume). Afterwards, samples were acidified by adding formic acid to 2% final volume, combined, and desalted using a C18 Sep-Pak (Waters, Milford, MA). The now combined sample was fractionated using basic pH reversed phase chromatography using a 1200 HPLC (Agilent; Santa Clara, CA) equipped with a UV-DAD detector and fraction collection system. Then, the resulting 12 fractions were desalted using the C18 StageTip procedure^14^. Each fraction was loaded onto a 100 µm id, 35 cm long column packed with 1.8 µm beads (Sepax, Newark DE) and separated using a 3 h gradient from 8–27% buffer B (99% acetonitrile and 1% formic acid) and buffer A (96% water, 3% acetonitrile and 1% formic acid) on an Easy 1000 nano-LC (Thermo-Fisher Scientific, San Jose, CA). All MS analyses were performed on an Orbitrap Fusion Lumos mass spectrometer (Thermo-Fisher Scientific, San Jose, CA) applying a multi-notch MS3 method^15,16^. The FA proteomics was performed as a service at the Thermo Fisher Center for multiplexed Proteomics at the Harvard Medical School.

The mass spectrometry proteomics data have been deposited to the ProteomeXchange Consortium via PRIDE^17^ partner repository with the dataset identifiers PXD011453^18^ (FA) and PXD010861^19^ (UUO).

### Bioinformatic analysis

#### mRNA-seq data

All samples were processed using an RNA-seq pipeline implemented in the bcbio-nextgen project (https://bcbio-nextgen.readthedocs.org). Raw reads were examined for quality issues using FastQC (http://www.bioinformatics.babraham.ac.uk/projects/fastqc/) to ensure library generation and sequencing were suitable for further analysis. Adapter sequences, other contaminant sequences (such as polyA tails and low quality sequences with PHRED quality scores less than five) were trimmed from reads using atropos^20^. Trimmed reads were aligned to UCSC build mm10 of the Mus musculus genome, augmented with transcript information from Ensembl release GRCm38.84 using STAR^21^. Alignments were checked for evenness of coverage, rRNA content, genomic context of alignments (for example, alignments in known transcripts and introns), complexity and other quality checks using a combination of FastQC, Qualimap^22^, MultiQC^23^ and custom code within the bcbio-nextgen pipeline. Counts of reads aligning to known genes were generated by featureCounts^24^. In parallel, Transcripts Per Million (TPM) measurements per isoform were generated by quasialignment using Salmon^25^. Normalization at the gene level was called with DESeq2^24–26^, preferring to use counts per gene estimated from the Salmon quasialignments by tximport^11,24–27^. The DEGreport Bioconductor package was used for QC and clustering analysis (https://bioconductor.org/packages/release/bioc/html/DEGreport.html). A Quality metrics report for UOO and FA sequencing data can be found on https://github.com/hbc/MouseKidneyFibrOmics/tree/master/reports.

#### Small RNA-seq data

All samples were processed using a small RNA-seq pipeline implemented in the bcbio-nextgen project (https://bcbio-nextgen.readthedocs.org/en/latest/). Quality control of the raw reads was performed as above for the RNA-seq data using FastQC and atropos. In the following, we focused on miRNA analysis but the small RNA seq dataset includes also t-RNAs and pi-RNAs.Trimmed reads were aligned to miRBase v21^28^ to the specific species with seqbuster^29^. In addition, the trimmed reads were aligned to the Mus musculus genome (version mm10) using STAR^21,29^. The aligned reads were analyzed with seqcluster^30^ to characterize the whole small RNA transcriptome and classify reads into rRNA, miRNA, repeats, genes, tRNAs and others from the UCSCannotation^31^. Finally, aligned reads were analyzed using miRDeep2^32^, an algorithm that assesses the fit of sequenced RNAs to a biological model of miRNA generation and correct folding. Alignments were checked for evenness of coverage, rRNA content, genomic context of alignments (for example, alignments in known transcripts and introns), complexity and other quality checks using a combination of FastQC, MultiQC^23^ and custom code within the bcbio-nextgen pipeline.

Data were loaded into R using the bcbioSmallRna R package (https://github.com/lpantano/bcbioSmallRna) and isomiRs Bioconductor package^33,34^ to get normalized expression values^13^.

#### Proteomics data

Raw data were converted to mzXML and searched via Sequest^35^ version 28 against a concatenated Uniprot^36^ database downloaded 02/04/2014. Variable modifications of oxidized methionine and over-labelling of TMT on serine, threonine and tyrosine were considered^37^. Mass tolerance parameters for peptide identification were ±25 ppm for precursor ions and ±0.9 Da for fragment ions. To distinguish forward and reverse hits, linear discriminant analysis^38^ was used and reverse hits were filtered to an FDR of 1% at the protein level. Using rules of parsimony shared peptides were collapsed into the minimally sufficient number of proteins (Table 1). Quantitation filters of >200 sum reporter ion S:N and >0.7 isolation specificity were incorporated. All abundance values were normalized with edgeR using TMM method^39^ and transformed the abundance values to log2 scale. UNIPROT ids were mapped to ensembl gene ids using the GRCm38.84 release to combine the proteomic data with the gene and miRNA expression data.

**Table 1:** Numerical description of protein data for UUO and FA.

Model	Peptides identified	Proteins identified	Proteins quantified
UUO	134010	10265	8942
FA	184278	9285	8417

#### Data processing

We used the normalized abundance values for each data type to populate the Rshiny app. To pair miRNA with genes, we used TargetScanHuman database^40^. Only pairs described in the database and pairs with the abundance correlation along time was lower than −0.7 were kept as valid miRNA-Gene pairs. This information is shown in the Rshiny app, at the bottom of the page, where the user can inspect filtered targets of miRNAs.

## Data Records

A list of all datasets per biological replicate is summarized in an experimental study table (Online-only Table 1).

GitHub as an easily accessible and widely used platform was used for our codes and QC reports. All raw data and processed data were uploaded to GEO and PRIDE and the code was finally archived at Zenodo, too^41^.

The raw sequencing data have been deposited in the GEO database with ID number GSE118341^42^.

The raw protein data have been deposited in the PRIDE database with ID numbers PXD011453^18^ and PXD010861^18,19^.

Gene expression estimates using salmon and tximport for the UUO model can be found in uuo_mrna.csv^11^.

Gene expression estimates using salmon and tximport for the FA model can be found in fa_mrna.csv^10^.

miRNA data resulting from seqbuster and isomiRs for the UUO model can be found in uuo_mirna.csv^10,13^.

miRNA data resulting from seqbuster and isomiRs for the FA model can be found in fa_mrna.csv^12^.

Protein data resulting from limma for the UUO model can be found in uuo_protein.csv^43^.

Protein data resulting from limma for the FA model can be found in fa_protein.csv^43,44^.

## Technical Validation

To assess the quality of the mRNA and small RNA sequencing libraries, basic quality metrics were summarized per sample in Tables 2 and 3 as well as in the supplementary information (Supplementary Tables 1 and 2). The full quality metrics report for UOO and FA sequencing data can be found on https://github.com/hbc/MouseKidneyFibrOmics/tree/master/reports. All mRNA-seq samples had a mapping rate >90%, ribosomal content <1% and exonic mapping rate >80%, showing a good enrichment of reads on coding genes. Small RNA-seq samples had 3′ adapter in more than 80% of the reads, a read size distribution of 22 after adapter removal. After removal of reads shorter than 18 nts, more than 50% of the reads mapped to miRNA sequences.

**Table 2:** Quality metrics of UUO mRNA Seq data.

Sample Name	Reads	rRNA	5′-3′ bias	M Aligned	Exon %
normal_1	40.5 M	0.30%	0.99	39.2	88.7
normal_2	29.7 M	0.20%	0.99	28.9	88.7
normal_3	32.4 M	0.20%	0.99	31.5	88.5
day3_4	37.4 M	0.40%	0.99	36.4	87.4
day3_5	34.9 M	0.70%	0.98	33.7	85.2
day3_6	34.6 M	1.50%	0.84	33.2	83.4
day3_7	39.4 M	0.30%	0.87	38	86.4
day7_8	37.4 M	0.30%	0.97	36.2	84.6
day7_9	39.5 M	0.40%	0.85	38.2	86.2
day7_10	38.8 M	0.40%	0.87	37.4	85.7
day7_11	26.7 M	0.30%	0.9	25.6	84.8
day14_12	30.1 M	0.30%	0.97	29.1	84.5
day14_13	17.0 M	0.20%	0.88	16.1	84
day14_14	23.6 M	0.40%	0.9	22.8	82.2
day14_15	24.7 M	0.40%	0.87	23.9	83.9

**Table 3:** Quality metrics of UUO small RNA seq data.

Sample Name	M Seqs	% with adapter	miRNAs	isomiRs
normal_1	21.1	98	593	17485
normal_2	22.9	98	646	21147
normal_3	26.9	98	650	20328
day3_1	22.6	99	775	27171
day3_2	21.2	99	799	28416
day3_3	19.1	99	789	27292
day3_4	22.8	98	784	28022
day7_1	23.8	99	801	28313
day7_2	19.4	99	710	22612
day7_3	27.3	99	804	30444
day7_4	23.6	99	794	27713
day14_1	25.4	99	770	28953
day14_2	21.5	99	741	25008
day14_3	29.4	99	785	28947
day14_4	21.2	99	730	34074

**Fig. 2:**
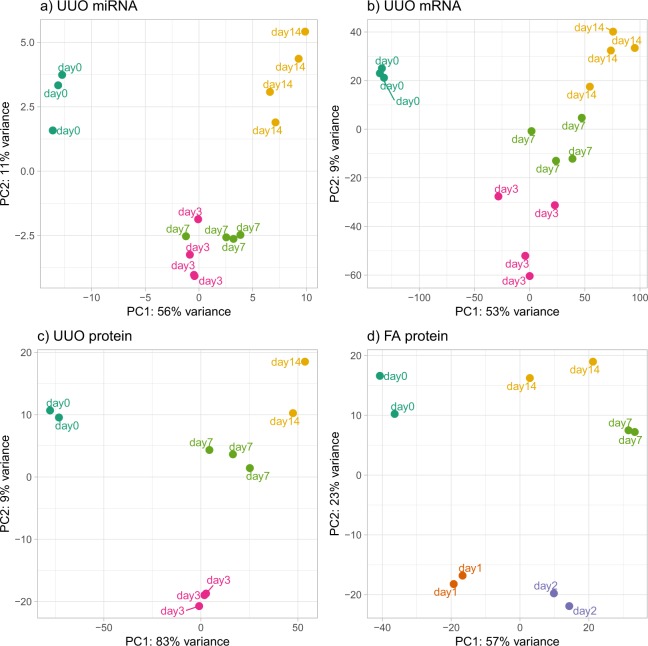
Principal component analysis (PCA) of all UUO datasets and FA proteins. The normalized expression abundance of mRNAs, proteins and miRNA was used. Each color represents a time point in the dataset. (**a**) miRNA expression in kidneys from the UUO model shows day 3 and 7 being in the same cluster, while the normal and the latest time points are distinct. (**b**) The greatest variation in gene expression in the UUO model is observed along the first principal component (PC) between normal and injured samples, with the second PC separating injury times. (**c**) A similar pattern is observed using UUO protein expression, with higher consistency within sample groups allowing for better discrimination between time points. (**d**) Protein expression in the FA model shows different clusters for each time point, PC1 separating normal from the injured samples, and PC2 separating early injury from later time points.

To further assess the quality of the data, we performed principal component analysis of the normalized gene and protein expression values to determine if the biological replicates show consistency and group by time after injury. Figure 2 shows strong clustering of the replicates and separation among time points for UUO miRNA (a), UUO mRNA (b), UUO protein (c) and FA protein (d). FA mRNA and miRNA data are shown in the supplementary information (Supplementary Fig. 1). The separation among time points is maximal in the protein datasets, while the mRNA and miRNA datasets show how day 7 and day 14 (for mRNA) and day 3 and day 7 (for miRNA) are more closely related. This difference can be explained by the fact that miRNA changes happen before mRNA changes, and the miRNA profile of day 3 could be impacting the day 7 mRNA profile. The same might be true for day 7 of the miRNA profile and day 14 of the mRNA profile.

**Fig. 3:**
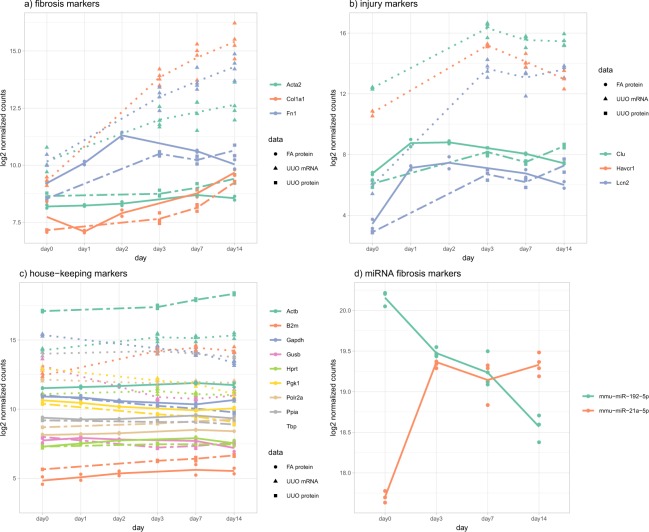
Expression profiles of fibrosis and injury markers and housekeeping genes. UUO mRNA (dotted line), UUO protein (dashed line), FA protein (standard line), and UUO miRNA (standard line). (**a**) The fibrosis markers α-smooth muscle actin (Acta2), collagen (Col1a1) and fibronectin (Fn1) show increasing expression over time for UUO mRNA, UUO protein and FA protein datasets. (**b**) Kidneyinjury markers clusterin (Clu), kidney injury molecule 1 (Kim-1 alias Havcr) and lipocalin-2 (Ngal alias Lcn2) show increased expression early on without further significant increases over time. (**c**) Nine commonly used housekeeping genes show no change of expression in all the datasets. (**d**) miRNAs miR-192 and -21 are involved in kidney pathogenesis and show expression changes over time for the UUO model.

Additionally, we have looked at the expression of known housekeeping genes and well-known fibrosis and injury markers (Fig. 3 and Supplementary Fig. 2) to evaluate the validity of the animal and omics experiments. The selection of these genes is purely based on literature and did not result from any statistical analysis. The coefficient of variation average for housekeeping genes is 3.3%, indicating stable expression in all kidney samples. Classical fibrosis markers α-smooth muscle actin (Acta2), collagen (Col1a1) and fibronectin (Fn1) are continuously increased over time for the irreversible UUO model whereas there is a decrease towards normal in the reversible FA model. Kidney injury markers clusterin (Clu), kidney injury molecule 1 (Kim-1 alias Havcr) and lipocalin-2 (Ngal alias Lcn2) are strongly increased early on without further significant increases over time in the UUO model. In the FA model, similar to fibrosis markers, injury markers indicate recovery at the later time points. Thus, fibrosis and injury marker profiles correspond to previously published data. miR-192, a kidney-enriched miRNA involved in regulation of the sodium transport, is decreased in de-differentiated fibrotic kidneys^45^ while miR-21 increased according to its role in fibrosis^46,47^.

## Usage Notes

Analyses of parts of this dataset have been published before in separate publications^4,5^ using the FA mRNA and miRNA data to identify new biomarkers of fibrosis and to find miRNAs involved in the pathophysiology, respectively. This bigger dataset here with mRNA, miRNA and protein data from two kidney fibrosis models could be utilized, among other possibilities, to (1) identify and validate new target genes or miRNAs for kidney fibrosis; (2) develop a more comprehensive understanding of the pathophysiology; (3) identify novel gene-miRNA regulatory networks related to kidney fibrosis; and (4) discover novel transcripts (genes and miRNAs) in fibrotic kidneys. The various ways to use and re-use proteomics data have been reviewed and well-stated elsewhere^48,49^. Furthermore, a large number of algorithms for differential gene expression analysis are available through the BioConductor project website to re-analyse or further investigate this dataset. In addition, we have developed a searchable web-tool for simple and quick inquiries related to this dataset: http://hbcreports.med.harvard.edu/fmm/.

Kidney fibrosis as well as any other fibrotic disease are complex and involve complementary changes in gene and protein expression as the disease initiates and progresses; thereby, affecting various signalling pathways^50^. Restricting data generation and analysis to a single omic dataset shows only one facet of this complex pathophysiology. Therefore, the value of using multi omics data lies in the generation of more representative multi-layered networks which can uncover causative changes^51^. Comparable approaches have been made for other fibrotic diseases either in one study^52^ or retrospectively by reviewing individually generated and published single omics datasets^53^. Similarly, individual omics datasets generated by others using human kidney disease samples^54^ or human fibrotic diseases could be combined and integrated with our dataset to explore translational aspects or “universal” fibrotic patterns. A plethora of different integration algorithms are available and could be used for this purpose^55^.

One main limitation in the data reported here is that bulk omics datasets do not distinguish among different kidney cell types and infiltrated immune cells; however, this bulk data could be useful for power calculations for designing future single-cell and omics studies.

## Supplementary Information

### ISA-Tab metadata file

Download metadata file

### Supplementary information

Supplementary Information

## Data Availability

The code used to process the data and perform the quality control and visualization analysis can be found at: https://github.com/hbc/MouseKidneyFibrOmics and at Zenodo^41^.

## Online-only Table

**Online-only Table 1:** Experimental study table.

Subjects	Protocol 1	Protocol 2	Protocol 3	Protocol 4	Data	Protocol 5	Data	Protocol 6	Protocol 7	Data
UUO mouse 1	no surgery	Kidney dissection	RNA extraction	mRNA Seq	GSE118341	small RNA seq	GSE118341	protein isolation	10-plex TMT LC/MS proteomics	PXD010861
UUO mouse 2	no surgery	Kidney dissection	RNA extraction	mRNA Seq	GSE118341	small RNA seq	GSE118341	protein isolation	10-plex TMT LC/MS proteomics	PXD010861
UUO mouse 3	no surgery	Kidney dissection	RNA extraction	mRNA Seq	GSE118341	small RNA seq	GSE118341			
UUO mouse 4	UUO	Kidney dissection after 3 days	RNA extraction	mRNA Seq	GSE118341	small RNA seq	GSE118341	protein isolation	10-plex TMT LC/MS proteomics	PXD010861
UUO mouse 5	UUO	Kidney dissection after 3 days	RNA extraction	mRNA Seq	GSE118341	small RNA seq	GSE118341	protein isolation	10-plex TMT LC/MS proteomics	PXD010861
UUO mouse 6	UUO	Kidney dissection after 3 days	RNA extraction	mRNA Seq	GSE118341	small RNA seq	GSE118341	protein isolation	10-plex TMT LC/MS proteomics	PXD010861
UUO mouse 7	UUO	Kidney dissection after 3 days	RNA extraction	mRNA Seq	GSE118341	small RNA seq	GSE118341			
UUO mouse 8	UUO	Kidney dissection after 7 days	RNA extraction	mRNA Seq	GSE118341	small RNA seq	GSE118341	protein isolation	10-plex TMT LC/MS proteomics	PXD010861
UUO mouse 9	UUO	Kidney dissection after 7 days	RNA extraction	mRNA Seq	GSE118341	small RNA seq	GSE118341	protein isolation	10-plex TMT LC/MS proteomics	PXD010861
UUO mouse 10	UUO	Kidney dissection after 7 days	RNA extraction	mRNA Seq	GSE118341	small RNA seq	GSE118341	protein isolation	10-plex TMT LC/MS proteomics	PXD010861
UUO mouse 11	UUO	Kidney dissection after 7 days	RNA extraction	mRNA Seq	GSE118341	small RNA seq	GSE118341			
UUO mouse 12	UUO	Kidney dissection after 14 days	RNA extraction	mRNA Seq	GSE118341	small RNA seq	GSE118341	protein isolation	10-plex TMT LC/MS proteomics	PXD010861
UUO mouse 13	UUO	Kidney dissection after 14 days	RNA extraction	mRNA Seq	GSE118341	small RNA seq	GSE118341	protein isolation	10-plex TMT LC/MS proteomics	PXD010861
UUO mouse 14	UUO	Kidney dissection after 14 days	RNA extraction	mRNA Seq	GSE118341	small RNA seq	GSE118341			
UUO mouse 15	UUO	Kidney dissection after 14 days	RNA extraction	mRNA Seq	GSE118341	small RNA seq	GSE118341			
FA mouse 1	no treatment	Kidney dissection	RNA extraction	mRNA Seq	GSE65267	small RNA seq	GSE61328	protein isolation	10-plex TMT LC/MS proteomics	PXD011453
FA mouse 2	no treatment	Kidney dissection	RNA extraction	mRNA Seq	GSE65267	small RNA seq	GSE61328	protein isolation	10-plex TMT LC/MS proteomics	PXD011453
FA mouse 3	no treatment	Kidney dissection	RNA extraction	mRNA Seq	GSE65267	small RNA seq	GSE61328			
FA mouse 4	single i.p. injection 250 mg/kg FA	Kidney dissection after 1 day	RNA extraction	mRNA Seq	GSE65267			protein isolation	10-plex TMT LC/MS proteomics	PXD011453
FA mouse 5	single i.p. injection 250 mg/kg FA	Kidney dissection after 1 day	RNA extraction	mRNA Seq	GSE65267			protein isolation	10-plex TMT LC/MS proteomics	PXD011453
FA mouse 6	single i.p. injection 250 mg/kg FA	Kidney dissection after 1 day	RNA extraction	mRNA Seq	GSE65267					
FA mouse 7	single i.p. injection 250 mg/kg FA	Kidney dissection after 2 days	RNA extraction	mRNA Seq	GSE65267			protein isolation	10-plex TMT LC/MS proteomics	PXD011453
FA mouse 8	single i.p. injection 250 mg/kg FA	Kidney dissection after 2 days	RNA extraction	mRNA Seq	GSE65267			protein isolation	10-plex TMT LC/MS proteomics	PXD011453
FA mouse 9	single i.p. injection 250 mg/kg FA	Kidney dissection after 2 days	RNA extraction	mRNA Seq	GSE65267					
FA mouse 10	single i.p. injection 250 mg/kg FA	Kidney dissection after 3 days	RNA extraction	mRNA Seq	GSE65267	small RNA seq	GSE61328	protein isolation	10-plex TMT LC/MS proteomics	PXD011453
FA mouse 11	single i.p. injection 250 mg/kg FA	Kidney dissection after 3 days	RNA extraction	mRNA Seq	GSE65267	small RNA seq	GSE61328	protein isolation	10-plex TMT LC/MS proteomics	PXD011453
FA mouse 12	single i.p. injection 250 mg/kg FA	Kidney dissection after 3 days	RNA extraction	mRNA Seq	GSE65267	small RNA seq	GSE61328			
FA mouse 13	single i.p. injection 250 mg/kg FA	Kidney dissection after 7 days	RNA extraction	mRNA Seq	GSE65267	small RNA seq	GSE61328	protein isolation	10-plex TMT LC/MS proteomics	PXD011453
FA mouse 14	single i.p. injection 250 mg/kg FA	Kidney dissection after 7 days	RNA extraction	mRNA Seq	GSE65267	small RNA seq	GSE61328	protein isolation	10-plex TMT LC/MS proteomics	PXD011453
FA mouse 15	single i.p. injection 250 mg/kg FA	Kidney dissection after 7 days	RNA extraction	mRNA Seq	GSE65267	small RNA seq	GSE61328			
FA mouse 16	single i.p. injection 250 mg/kg FA	Kidney dissection after 14 days	RNA extraction	mRNA Seq	GSE65267	small RNA seq	GSE61328	protein isolation	10-plex TMT LC/MS proteomics	PXD011453
FA mouse 17	single i.p. injection 250 mg/kg FA	Kidney dissection after 14 days	RNA extraction	mRNA Seq	GSE65267	small RNA seq	GSE61328	protein isolation	10-plex TMT LC/MS proteomics	PXD011453
FA mouse 18	single i.p. injection 250 mg/kg FA	Kidney dissection after 14 days	RNA extraction	mRNA Seq	GSE65267	small RNA seq	GSE61328			
