# Supplementary material for: Multi omics analysis of fibrotic kidneys in two mouse models
Source: Sci Data. 2019 Jun 14;6:92. doi: 10.1038/s41597-019-0095-5 (PMC6570759; doi:10.1038/s41597-019-0095-5)
Supplement: Supplementary file 2 — Supplementary Information [file 41597_2019_95_MOESM2_ESM.pdf]

Supplementary Information

Multi Omics analysis of fibrotic kidneys in two mouse models

Mira Pavkovic[1,2*], Lorena Pantano[3,*], Cory V. Gerlach[1,2,4], Sergine Brutus[1,4,5], Sarah A. Boswell[1], Robert A. Everley[1], Jagesh Shah[1,2], Shannan H Sui[3], Vishal S. Vaidya[1,2,4]

1. Laboratory of Systems Pharmacology, Harvard Medical School, Boston, MA
2. Department of Medicine – Renal Division, Brigham and Women's Hospital, Boston, MA
3. Bioinformatics Core, Harvard T.H. Chan School of Public Health, Boston, MA
4. Department of Environmental Health, Harvard T. H. Chan School of Public Health, Boston, MA
5. Department of Systems Biology, Harvard Medical School, Boston, MA

* co-first

corresponding author: Vishal S. Vaidya (vvaidya@partners.org)

Contents

Supplementary Figure 1 Principal component analysis (PCA) of previously published data for FA mRNA and miRNA.. 2

Supplementary Figure 2 Expression profiles of fibrosis and injury markers and housekeeping genes from previously published FA mRNA and miRNA data. 3

Supplementary Table 1. Quality metrics of FA mRNA Seq data 4

Supplementary Table 2. Quality metrics of FA small RNA Seq data 5

Supplementary Figure 1 Principal component analysis (PCA) of previously published data for FA mRNA and miRNA. Raw data was re-analyzed and the normalized expression abundance of mRNAs and miRNAs was used. Each color represents a time point in the dataset. a) miRNA and b) mRNA expression in the FA model clusters for each time point, with PC1 separating normal from post injury groups, and PC2 separating the samples according to time points.

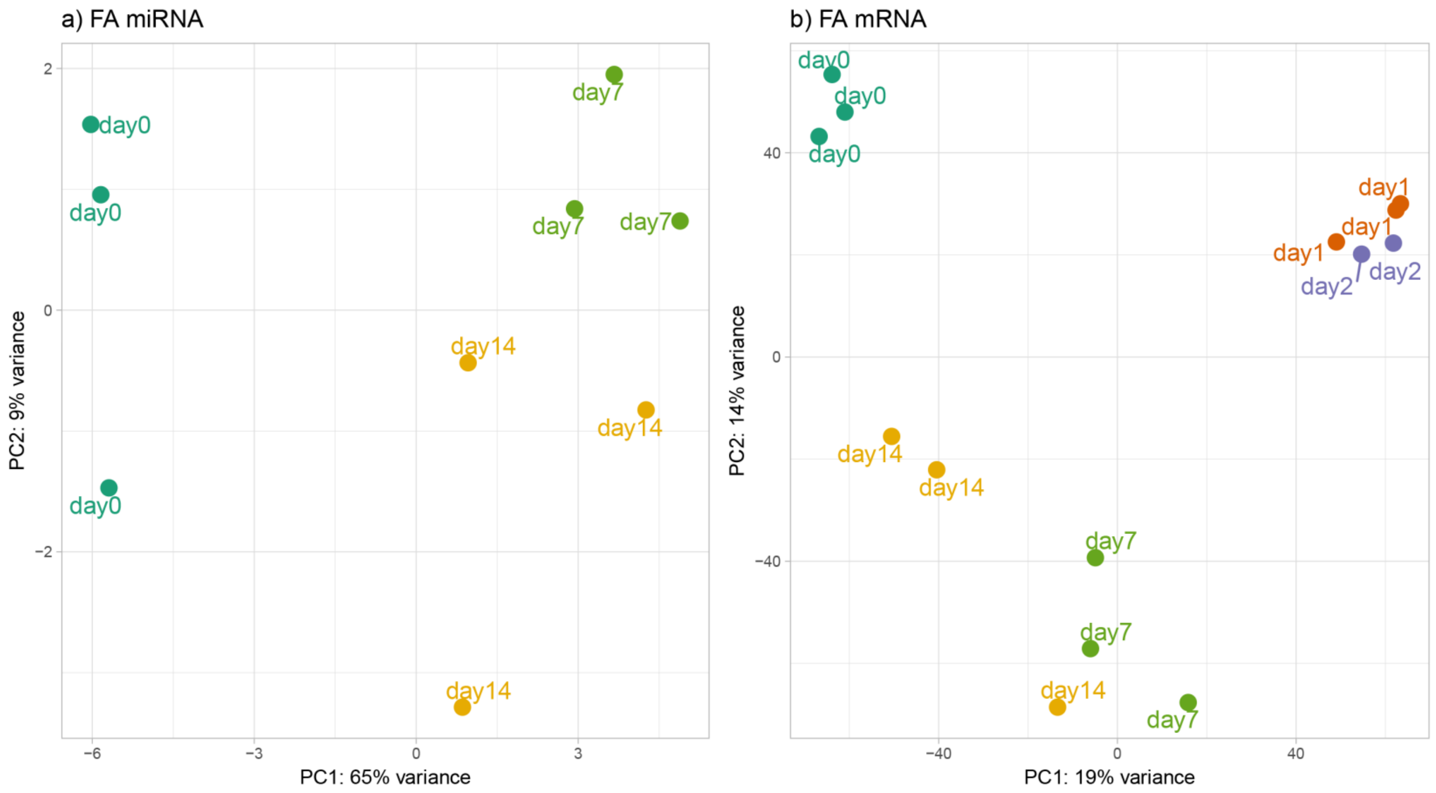

Supplementary Figure 2 Expression profiles of fibrosis and injury markers and housekeeping genes from previously published FA mRNA and miRNA data. Raw data was re-analyzed and mRNA expression was plotted for a) fibrosis and b) injury markers, and c) housekeeping genes, as well as miRNA expression for d) miR-192 and -21.

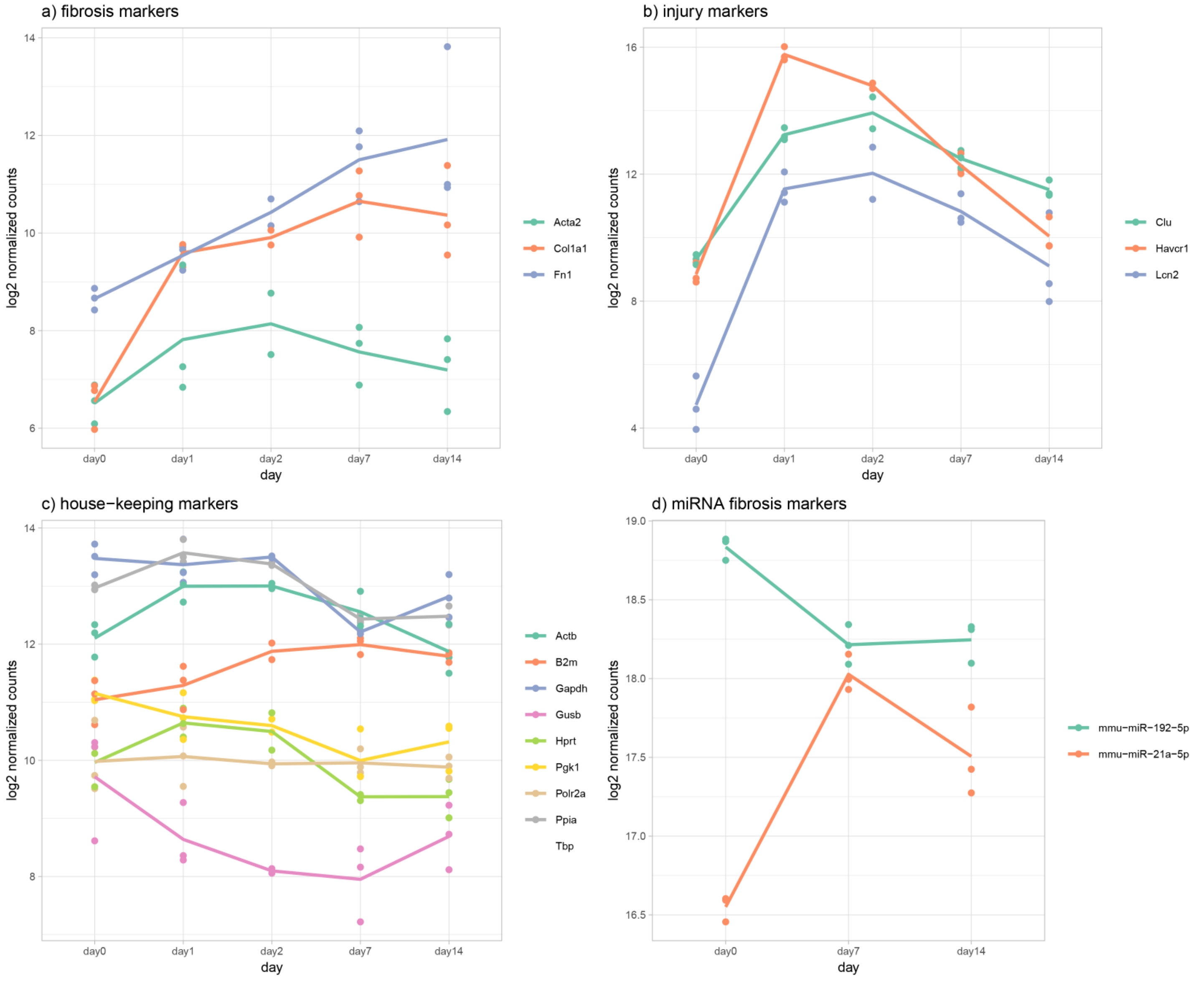

Supplementary Table 1. Quality metrics of FA mRNA Seq data

Sample Name	Reads	rRNA	5'-3' bias	M Aligned	Exon %
**normal_1**	97	0.6101	0.91	59	73.06
**normal_2**	178	0.5805	0.79	123	71.4
**normal_3**	76	0.6432	0.94	53	78.35
**day1_1**	88	0.5913	0.68	67	73.44
**day1_2**	152	0.5996	0.63	126	72.9
**day1_3**	165	0.5923	0.55	141	71.71
**day2_1**	179	0.5505	0.6	122	66.76
**day2_2**	63	0.5663	0.9	21	75.18
**day2_3**	85	0.5601	0.72	68	63.63
**day3_1**	97	0.5297	0.73	74	60.22
**day3_2**	78	0.5077	0.68	58	63.1
**day3_3**	121	0.5685	0.73	94	65.26
**day7_1**	81	0.5552	0.56	67	65
**day7_2**	74	0.5554	0.76	59	64.09
**day7_3**	112	0.5837	0.66	68	65.24
**day14_1**	87	0.6	0.66	57	70.97
**day14_2**	104	0.6463	0.74	72	74.43
**day14_3**	80	0.5952	0.86	62	63.33

Supplementary Table 2. Quality metrics of FA small RNA Seq data

Sample Name	M Seqs	% with Adapter	miRNAs	isomiRs
**A196_normal**	6.9	99	525	11305
**A200_normal**	8.4	99	557	13686
**A201_normal**	6.8	99	504	10677
**A178_day3**	7.9	98	594	12870
**A74_day3**	7.8	97	586	13474
**A75_day3**	6.6	97	549	11057
**A180_day7**	10.1	99	623	15538
**A181_day7**	7.0	99	553	11689
**A80_day7**	7.8	97	562	13506
**A84_day14**	8.1	99	568	14134
**A85_day14**	6.9	98	513	10462
**A189_day14**	6.7	94	562	12164
